# Exploring a Possible Link Between Tinnitus and the Risk of Obstructive Sleep Apnea—A National Population-Based Cohort Study Using Propensity Score Matching Analysis

**DOI:** 10.3390/jcm14217492

**Published:** 2025-10-23

**Authors:** Seung Jae Lee, Song I Park, Ick Soo Choi, Hyun Jin Lee, Jeon Mi Lee

**Affiliations:** 1 Department of Otorhinolaryngology-Head and Neck Surgery, Ilsan Paik Hospital, Inje University College of Medicine, 170, Juhwa-ro, Ilsanseo-gu, Goyang 10380, Gyeonggi-do, Republic of Korea; 2 Department of Otorhinolaryngology-Head and Neck Surgery, Incheon St. Mary’s Hospital, College of Medicine, The Catholic University of Korea, Seoul 21431, Republic of Korea

**Keywords:** tinnitus, obstructive sleep apnea, sleep disturbance, STOP-BANG questionnaire, hearing loss

## Abstract

**Objectives:** The association between tinnitus and obstructive sleep apnea (OSA) has received attention; however, the mechanisms linking both conditions with diverse outcomes are still unclear. This study aimed to evaluate the association between OSA risk and tinnitus, including the OSA-related characteristics affecting tinnitus. **Methods:** We included participants aged 40–65 years with auditory examinations and STOP-BANG questionnaire between 2019 and 2021 from the Korean National Health and Nutrition Examination Survey data. Possible causative factors for tinnitus were identified using logistic regression analysis. Participants in the low- and high-risk groups for OSA were 1:1 matched using propensity score matching to compare the possible causative factors. **Results:** The high-risk group exhibited a higher incidence of tinnitus, persistence, and severity. However, when the causative factors of tinnitus were matched, no significant differences were observed. The most likely contributing factors were high-frequency hearing level in the worse ear for the experience and persistence of tinnitus and smoking for the severity of tinnitus. The high-risk group experienced tinnitus more often than the low-risk group, with no differences between the groups when tinnitus-affective factors were controlled. **Conclusions:** These findings suggest that tinnitus is unaffected by OSA itself but by the hearing loss that accompanies OSA.

## 1. Introduction

Tinnitus is a common otologic symptom where an individual perceives sound in the absence of external auditory stimulus [1]. Studies showed that 14% of adults experience tinnitus, and 1–2% of patients with tinnitus complain of severe tinnitus [2,3]. Similarly, approximately 20.7% Korean adults have tinnitus [4]. This high tinnitus prevalence shows an increasing trend with age regardless of sex [5]. Since tinnitus increases with age, as does the prevalence of age-related hearing loss, previous studies have proposed several mechanisms linking the two conditions, mainly involving peripheral auditory deafferentation and deficiencies in the noise-cancellation pathway [1,6]. In addition, central mechanisms such as maladaptive cortical reorganization and increased neural synchrony in the auditory cortex have been suggested to contribute to chronic tinnitus after hearing loss [3,6]. However, its exact pathophysiology remains unknown.

Several factors have been proposed as potential causes of tinnitus, including age, sex, hearing level, lifestyle (smoking and alcohol consumption), and comorbidities (depression, diabetes, hypertension, migraine) [7]. Although the results may differ depending on the study population or analysis method, a consensus exists that some factors are closely related to tinnitus.

Sleep disturbance, reported among 50–75% of patients with chronic tinnitus, has been recently identified as a possible cause of tinnitus [8]. Obstructive sleep apnea (OSA) is a common sleep-related disorder causing repetitive complete or partial collapse of the upper respiratory airway, leading to decreased blood oxygen saturation. It has a prevalence of approximately 4–6% globally, making it a relatively common sleep disorder [9]. Patients with OSA experience various night-time symptoms, such as snoring, hypopnea or apnea, arousal responses, and nocturia, and various daytime symptoms, including sleepiness, fatigue, depression, increased risk of cardiovascular sequelae, impaired work performance, and decreased quality of life [10]. Having emphasized OSA’s influence, the Korean National Health and Nutrition Examination Survey (KNHANES) implemented a STOP-BANG questionnaire (SBQ) since 2019, developed to assess OSA’s risk [11]. This questionnaire provides superior reliability and accuracy in identifying individuals at risk of OSA compared with the existing Berlin questionnaire or Epworth sleepiness scale [12].

With the increasing interest in the association between tinnitus and OSA, some studies have suggested that OSA may have a significant relationship with tinnitus. The most common hypothesis is that sleep apnea causes hearing loss, ultimately causing tinnitus [13]. Other studies have hypothesized that snoring noise caused by OSA can itself cause tinnitus [14], and have argued that OSA causes tinnitus by worsening sleep quality [15]. Although the proposed hypotheses are specific and reasonable, no consistent results exist regarding the relationship between tinnitus and OSA.

Therefore, this study aimed to identify the association between tinnitus and OSA risk, and to determine which aspects of OSA affect tinnitus. To the best of our knowledge, this study included the largest patient cohort. Additionally, propensity score matching (PSM) analysis can effectively control for confounders and provide accurate comparisons.

## 2. Materials and Methods

### 2.1. Study Design and Participants

This cross-sectional study used data from the KNHANES conducted by the Korean Disease Control Headquarters. A cohort was surveyed from 2019–2021, after sampling the entire Korean population using a two-stage clustered (regional and household) stratified random sampling method. Every year, 7000–10,000 individuals in approximately 3500 households were selected from a panel to represent the population.

The study included individuals aged between 41 and 65 years with completed auditory examinations using a pure-tone audiogram (PTA) and SBQ. Participants with missing SBQ, PTA, tinnitus, or covariate data were excluded. Participants were categorized into low-, intermediate-, and high-risk groups for OSA per the SBQ, and only the low- and high-risk groups were enrolled in the final analysis. We excluded those with an intermediate risk to achieve a clearer distinction between the groups. This study was approved by the Institutional Review Board of the Inje University Ilsan Paik Hospital (approval number: 2024-09-008). Written informed consents were waived due to the retrospective design of the study. The study was performed in accordance with the tenets of the Declaration of Helsinki and Good Clinical Practice guidelines.

### 2.2. STOP-BANG Questionnaire (OSA Risk Evaluation)

OSA risk was evaluated using the SBQ, which included eight dichotomous questions: loud snoring (S), daytime fatigue (T), observed apnea (O), high blood pressure (BP) (P), body mass index (BMI) > 30 kg/m^2^ (B), age > 50 years (A), neck circumference (NC) > 40 cm (N), and male sex (G) [11]. A low-risk group was defined as those who answered “yes” to 0–2 questions, the intermediate-risk group as those who answered “yes” to 3–4 questions, and the high-risk group as those who answered “yes” to 5–8 questions or those who answered “yes” to two or more STOP questions with BMI > 30 kg/m^2^, NC > 40 cm, or male sex. As described above, only participants categorized into low- and high-risk groups were enrolled in the final analysis to achieve a clearer distinction between the risk groups, excluding those in the intermediate-risk group.

### 2.3. Audiometric Evaluation

PTA was performed in a soundproof booth using an automatic audiometer (SA 203; Entomed, Sweden) to determine the air-conduction hearing thresholds at 0.5, 1, 2, 3, 4, and 8 kHz in both ears. The order of the sound frequencies was randomly evaluated [16]. The mean hearing threshold was calculated bilaterally as the mean decibel at 0.5, 1, 2, and 4 kHz, and the extra-high frequency average was defined as the mean threshold at 4 and 8 kHz.

### 2.4. Tinnitus Evaluation

Participants were surveyed using three tinnitus-related questions. First, they were asked if they had experienced or heard any ringing, buzzing, roaring, or hissing sounds without an external acoustic source for more than 5 min in the past. The response options were ‘‘Yes,” “No,’’ or ‘‘I cannot remember.’’ Then, if the participants answered to “Yes,” the second question was asked if tinnitus persisted over 6 months, and the response was also divided into “Yes” or “No.” The final question concerned the subjective distress level of tinnitus: “Are these sounds bothersome to you?” Responses were rated only for this question measured and evaluated using a numeric rating scale (NRS) ranging from 0–10, with 0 being the lowest and 10 the highest distress level.

### 2.5. Covariates

Patients’ demographic characteristics included age, sex, education level, marital status, and subjective health perception. Baseline comorbidities, including BMI, hypertension (HTN), hypercholesterolemia, hypertriglyceridemia, anemia, masticatory difficulties, asthma, thyroid diseases, depression, otitis media, and renal diseases. Other health-related conditions, including alcohol consumption per month, average sleep time per day, and smoking history, were also identified using the KNHANESs.

### 2.6. Statistical Analysis

All statistical analyses were performed using SPSS version 21 for Windows (IBM Corp., Armonk, NY, USA). Sample weights were applied in all analyses to reflect the national population estimates. Means and standard deviations were used for the descriptive statistics. Variables were compared between the low- and high-risk groups using t-test for continuous variables and Pearson’s chi-squared test for categorical variables. Univariate and multivariate analyses were performed using a logistic regression model to examine the possible tinnitus causative factors. Variables implicated (*p* < 0.2) in univariate analysis were entered into the multivariate analysis model. PSM was used to identify OSA’s effects on tinnitus. Next, the participants in the low- and high-risk groups were 1:1 matched for possible causative factors and compared. A *p*-value < 0.05 was considered statistically significant.

## 3. Results

### 3.1. Study Population, Demographics, and Clinical Characteristics per OSA Risk

A total of 8,549 individuals aged 41–65 years participated in the survey between 2019–2021. After excluding those with missing data, 1,869 participants were enrolled and categorized into low- (*n* = 861), intermediate- (*n* = 699), and high-risk (*n* = 309) groups according to SBQ scores. We included only those in the low- and high-risk groups; thus, a total of 1170 participants were included in this study.

Demographic and clinical characteristics according to the OSA risk are presented in Table 1. Mean SBQ score was 1.53 ± 0.63 and 5.14 ± 1.05 (*p* < 0.001) for the low- and high-risk groups, respectively. The mean age was 50.62 ± 7.06 and 53.34 ± 6.84 years old for the low- and high-risk groups, respectively, significantly higher for the high-risk group (*p* < 0.001). Other factors affecting OSA were compared based on the OSA risk. Age, sex, marriage status, subjective health perception, BMI, HTN, hypercholesterolemia, hypertriglyceridemia, anemia, and smoking were shown to have a significant difference, while education level, alcohol consumption, average sleep time, masticatory difficulties, asthma, thyroid disease, depression, otitis media, and renal disease were not significantly different between both groups.

### 3.2. Hearing Levels per the OSA Risk

Hearing levels between the groups were analyzed according to the OSA risk. We compared the PTA from each ear and divided them into better and worse hearing sides to exclude the effects of asymmetrical hearing loss or other types of hearing loss (i.e., conductive or mixed hearing loss) in those with pathologically deteriorated hearing levels. The hearing levels of the high-risk group, evidenced by the average hearing thresholds of 0.5, 1, 2, and 4 kHz and thresholds within high frequencies (4 and 8 kHz) of the better-hearing sides, were significantly worse than those of the low-risk group (Table 2).

### 3.3. Tinnitus Levels per the OSA Risk

Answers to the three tinnitus-related questions were analyzed. Regarding tinnitus experience, 68 of 861 participants (7.9%) from the low-risk group and 36 of 309 participants (11.7%) from the high-risk group answered that they had experienced at least 5 min of tinnitus in the past, showing no statistical difference between the groups (*p* = 0.061). Regarding tinnitus persistence, 59 low-risk participants (6.9%) and 33 high-risk participants (10.7%) answered that they had experienced tinnitus for over 6 months, with significantly higher difference in the high-risk group (*p* = 0.043). A significant difference was also found in the tinnitus severity, which was 0.3 ± 1.2 and 0.5 ± 1.6 in NRS, respectively (*p* = 0.036). However, when the number of participants rated higher than an NRS of 7 was compared, it was 10 (1.2%) in the low-risk group and seven (2.3%) in the high-risk group, with no statistical differences (*p* = 0.171, Table 2).

### 3.4. Factors Associated with Tinnitus

While participants in the high-risk group exhibited a higher rate of tinnitus experience, persistence, and severity, we attempted to determine whether this phenomenon was due to factors other than OSA. We investigated the factors that may affect tinnitus and re-evaluated the answers to tinnitus-related questions after controlling for them.

Logistic regression analysis revealed tinnitus-associated factors (Table 3, Supplementary Table S1). Perceived stress, smoking, depression history, and high-frequency hearing levels in the worse ear significantly affected the experience of tinnitus. For tinnitus persistence over 6 months, smoking and high-frequency hearing levels in the worse ear were implicated. Smoking and depression history were factors affecting tinnitus severity.

### 3.5. Effect of OSA Risk on Tinnitus After Controlling Other Factors

We analyzed the effect of OSA risk on tinnitus by controlling for factors affecting tinnitus using a 1:1 PSM method (Table 4). Regarding tinnitus experience, we revealed that perceived stress, smoking, depression history, and high-frequency hearing levels in the worse ear were the most common causative factors. We created a model by adjusting for all four factors. In total, 309 participants from each group were matched and compared. When these four factors were controlled for, no significant difference was observed in tinnitus experience between both groups. We then created another four models by adjusting each of the four factors to identify the most influential factor. Perceived stress was adjusted in the first model, the second model was adjusted for smoking, the third model was adjusted for depression history, and the fourth model was adjusted for high-frequency hearing level in the worse ear. Each model included 309 participants. The difference between both groups disappeared when the high-frequency hearing level in the worse ear was controlled.

Smoking and high-frequency hearing levels in the worse ear were associated with tinnitus persistence. When these two factors were controlled for, the differences disappeared. Two additional models were created to identify the most effective factors. The first model was adjusted for smoking and the second model was adjusted for high-frequency hearing levels in the worse ear. The difference between both groups disappeared when the high-frequency hearing level in the worse ear was controlled.

Finally, smoking and depression history were found to affect tinnitus severity. When these two factors were controlled for, the differences between the groups disappeared. Two additional models were created in a similar manner. It was found that the difference between both groups disappeared when smoking was controlled, whereas it remained when only depression history was controlled.

## 4. Discussion

Our main findings can be summarized as follows. First, patients with higher OSA risk experienced tinnitus more frequently and in a more bothersome manner than those without OSA. Second, when the factors that affected tinnitus were controlled, no statistically significant differences existed in the experience, persistence, and severity of tinnitus between low- and high-risk OSA groups. Third, the most likely contributory factors are a high-frequency hearing level in the worse ear for the existence and persistence of tinnitus and smoking for the severity of tinnitus.

Various studies have been conducted on the relationship between OSA and tinnitus, with varying results. Although no clear causal relationship or pathophysiology has been identified, they are probably associated. These diverse results are due to the small number of cohorts; different methods of defining OSA, hearing, or tinnitus levels; difficulty in obtaining accurate control groups, and the extent to which the causative factors were adjusted. Addressing these issues, we used the SBQ to establish the low- and high-risk OSA group in a large national cohort. We then conducted a 1:1 PSM by adjusting for variables known to affect tinnitus. Although overnight polysomnography (PSG) is the gold standard for diagnosing OSA, its high cost and inconvenience limited participation. Furthermore, selecting a control group is challenging because patients without sleep apnea symptoms rarely undergo PSG. Therefore, in this study, the risk of OSA was assessed using the SBQ, a validated screening tool used to stratify OSA risk. The SBQ has demonstrated high screening sensitivity for identifying individuals at risk of OSA (83.6–100.0%) [17,18,19]. Its effectiveness in identifying individuals at risk of OSA is superior to that of other screening surveys, such as the Berlin questionnaire or Epworth sleepiness scale [12]. In this study, the SBQ effectively classified a large cohort into low- and high-risk groups for OSA, enabling balanced comparisons after matching.

In this study, 7.9% of the low-risk group and 11.7% of the high-risk group reported experiencing tinnitus for more than 5 min in the past, comparable with a recent study that reported 11.23% of 239.7 million American adults [20]. Although not statistically significant, tinnitus experience was slightly more frequent in the high-risk group, but this difference diminished after adjusting for possible causative factors. Nevertheless, it is challenging to determine whether this question accurately reflects the true experience of tinnitus. Tinnitus is commonly described as a phenomenon in which an individual perceives sound in the absence of an external auditory stimulus, but it should be distinguished from transient ear noise and last for at least 5 min [21]. Furthermore, if such a phenomenon occurs only occasionally, it can be differentiated from pathological tinnitus [22]. Therefore, it is difficult to ascertain the actual prevalence of tinnitus based solely on the first question, which may explain the lack of significant differences between the groups. The second question asked those who answered yes to the first question whether their tinnitus persisted for over 6 months, seeking to determine chronic tinnitus prevalence. We found that 6.9% of the low-risk group and 10.7% of the high-risk group had chronic tinnitus. The definition of chronic tinnitus usually refers to 1 to 3 months [21,23,24], and this question may have lowered chronic tinnitus prevalence in this study. Nevertheless, a meaningful difference existed between both groups in that chronic tinnitus was more prevalent in the high-risk group. The difference was also reduced when the causative factors were controlled. It also applied to tinnitus severity. The high-risk group showed a higher tinnitus severity, and the difference reduced after controlling for these factors. Notably, the mean tinnitus distress level was relatively low in both groups, which may be explained by the community-based characteristics of the KNHANES population including individuals with diverse tinnitus severity. Nonetheless, some participants still reported noticeable distress, indicating that tinnitus may have a meaningful, though generally modest, impact on daily life in this population.

While we found that the differences between both groups were reduced after identifying possible causative factors, we tried to identify which factor was the most effective. According to the analysis, the experience and persistence of tinnitus were most affected by high-frequency hearing levels in the worse ear, whereas tinnitus severity was most affected by smoking. The association between hearing loss and tinnitus is well established, and its effects on tinnitus are expected. Notably, ex-smokers and current smokers were found to have decreased tinnitus experience, persistence, and severity. However, previous studies have shown unexpected results, such as the preventive effects of high-dose caffeine intake or high alcohol consumption on tinnitus [7,25]. They hypothesized that caffeine enhances neurotransmission in both peripheral and central auditory pathways and improves auditory processing and emotional stability, thereby exerting a preventive effect on tinnitus [26,27,28]. Although direct evidence for a protective role of nicotine against tinnitus is limited, some studies have suggested that nicotinic acetylcholine receptor modulation can normalize tinnitus-related inhibitory imbalance or enhance auditory processing in animal and human models [29,30]. Both caffeine and nicotine act as central nervous system stimulants that may transiently modulate auditory pathway excitability. This shared mechanism may explain the reduced tinnitus indices observed among smokers in the present study, although further research is needed to clarify nicotine’s precise role.

Although we found an association between hearing level and tinnitus, the association between hearing level and OSA remains questionable. OSA can induce hearing loss through several mechanisms. A reduction in blood oxygen saturation in the auditory pathway could potentially affect the auditory function in patients with severe OSA [31]. OSA deteriorates the circulation surrounding the human cochlea, supplied by a single terminal artery, which lacks an alternative collateral blood supply [32]. Additionally, the reflex effects of hypoxia, hypercapnia, and increased sympathetic activity secondary to alterations in blood pressure during apnea episodes may cause deleterious cerebrovascular events, resulting in ischemic cochlear damage and permanent sensorineural hearing loss [33]. Conversely, Lee et al. argued that no differences occurred in hearing levels between those with and without OSA when age and sex were adjusted [34]. Although not shown in this study, we compared the hearing levels between the low- and high-risk groups while controlling for age and sex, with no statistical difference. We showed that the high-risk group exhibited more hearing loss than the low-risk group, but it is unknown whether the cause of hearing loss was the higher risk of OSA or the influence of other factors, such as age and sex.

Our study has some limitations. First, although the PSM analysis may have minimized the effects of various factors, this study did not identify and evaluate the exact onset time and duration of tinnitus and hearing loss, and OSA was not clinically diagnosed but assessed based on risk. These conditions usually do not have a clear onset time and develop over a long period, making it difficult to diagnose using current cross-sectional data. In addition, although the SBQ has high screening sensitivity, it is not a tool for diagnosing OSA. Hence, only a partial association between tinnitus and OSA can be suspected and not a causal relationship. Second, PTA within extra-high frequencies (>8 kHz), not included in this study protocol, could have affected tinnitus or OSA. Considering that patients with tinnitus and normal hearing often present with an increased threshold in the extra-high frequencies, which best represent localized, hidden cochlear damage [35], PTA or otoacoustic emission tests within extra-high frequencies could measure the cochlear functions of hidden extra-high frequency areas and predict the unknown relationship between the generation of tinnitus and OSA. Third, this study lacked comparisons and analyses between tinnitus severity and objective sleep parameters, measurable using nocturnal PSG. In a prospective study by Weingarten et al. [36], objective sleep parameters such as sleep efficiency, total sleep time, sleep onset latency, wake after sleep onset, number of awakenings, number of total arousals, number of spontaneous arousals, and the apnea-hypopnea index were not significantly correlated with tinnitus severity. Future research that monitors changes in tinnitus handicap inventory or objective sleep parameters before and after treatment for tinnitus or OSA would help clarify the causal link between tinnitus and OSA. Finally, the use of KNHANES dataset precluded the exclusion of specific otologic diseases such as chronic otitis media or vestibular schwannoma. However, the presence of such diseases is unlikely to have substantially affected our findings, as these conditions generally influence tinnitus indirectly through hearing deterioration rather than acting as independent causes of tinnitus. Nevertheless, we acknowledge that the inability to perform diagnosis-based exclusion remains a limitation of this study. However, despite these limitations, this study sheds light on the first analysis of the association between tinnitus and OSA risk, incorporating PSM analysis that controls for multiple factors and utilizes the most recent large-scale national database.

## 5. Conclusions

Taken together, this study provides valuable insights into the complex relationship between tinnitus and OSA risk. While the study found an association between the two conditions, the influence of other factors, such as hearing loss, smoking, and depression, cannot be overlooked. Future research should delve deeper into the underlying mechanisms and explore potential interventions to alleviate tinnitus in individuals with OSA. By addressing these factors and improving sleep quality, it may be possible to mitigate the impact of tinnitus and enhance the quality of life for affected individuals.

## Figures and Tables

**Table 1:** Covariates affecting OSA according to the OSA risk.

		Low-Risk Group (%)	High-Risk Group (%)	*p*-Value
Number of total participants		861	309	0.813
Participants according to each year	2019	203 (23.6)	70 (22.7)	
	2020	308 (35.8)	131 (42.4)	
	2021	350 (40.7)	108 (35.0)	
STOP-BANG score		1.53 ± 0.63	5.14 ± 1.05	<0.001 *
Age		50.62 ± 7.06	53.34 ± 6.84	<0.001 *
Sex (male:female)		612:249	304:5	<0.001 *
Education level (=graduated)	Elementary school	48 (5.6)	23 (7.4)	0.464
	Middle school	66 (7.7)	24 (7.8)	
	High school	351 (40.8)	113 (36.6)	
	College	396 (46.0)	149 (48.2)	
Marriage status (not married)		99 (11.5)	18 (5.8)	0.006 *
Subjective health perception	Good	284 (33.0)	63 (20.4)	<0.001 *
	Normal	472 (54.8)	178 (57.6)	
	Poor	105 (12.2)	68 (22.0)	
BMI		23.77 ± 2.84	27.58 ± 3.91	<0.001 *
HTN	Normal	467 (54.2)	43 (13.9)	<0.001 *
	Pre-HTN	304 (35.3)	61 (19.7)	
	HTN	90 (10.5)	205 (66.3)	
Hypercholesterolemia (=yes)		205 (23.8)	140 (45.3)	<0.001 *
Hypertriglyceridemia (=yes)		164 (19.0)	93 (30.1)	<0.001 *
Anemia (=yes)		45 (5.2)	7 (2.3)	0.045 *
Alcohol consumption	None	16 (1.9)	7 (2.3)	0.085
	More than once per month	511 (59.3)	204 (66.0)	
	Less than once per month	334 (38.8)	98 (31.7)	
Average sleep time		7.29 ± 6.39	6.67 ± 4.94	0.079
Smoking	None	59 (6.9)	9 (2.9)	0.006 *
	Ex-smoker	434 (50.4)	182 (58.9)	
	Current smoker	368 (42.7)	118 (38.2)	
Masticatory difficulties (=yes)		188 (21.8)	69 (22.3)	0.92
Asthma (=yes)		20 (2.3)	11 (3.6)	0.340
Thyroid disease (=yes)		17 (2.0)	7 (2.3)	0.940
Depression (=yes)		46 (5.3)	8 (2.6)	0.069
Otitis media (=yes)		43 (5.0)	20 (6.5)	0.401
Renal disease (=yes)		8 (0.9)	3 (1.0)	1.000

Abbreviations: BMI, body mass index; HTN, hypertension. * Statistically significant at *p* < 0.05.

**Table 2:** Hearing and tinnitus levels between the groups according to the OSA risk.

Hearing Threshold (dB)	Low-Risk Group	High-Risk Group	*p*-Value
Mean hearing threshold (better ear)	13.58 ± 8.93	15.83 ± 9.85	<0.001 *
Mean hearing threshold (worse ear)	18.96 ± 11.43	22.01 ± 12.56	<0.001 *
Mean high-frequency hearing threshold (better ear)	19.99 ± 13.75	24.63 ± 14.82	<0.001 *
Mean high-frequency hearing threshold (worse ear)	28.00 ± 16.45	34.08 ± 17.95	<0.001 *
**Tinnitus**	**Low-risk** **Group** **(N, %)**	**High-risk** **Group** **(N, %)**	***p*** **-value**
Tinnitus experience (=yes)	68 (7.9)	36 (11.7)	0.061
Tinnitus persistence (more than 6 months) (=yes)	59 (6.9)	33 (10.7)	0.043 *
Subjective distress level (mean NRS)	0.29 ± 1.19	0.50 ± 1.58	0.036 *
NRS ≥ 7	10 (1.2)	7 (2.3)	0.171

Abbreviations: NRS, numerical rating scale. * Statistically significant at *p* < 0.05.

**Table 3:** Logistic regression analysis of factors associated with tinnitus.

Variables		Number (%)	Experience of Tinnitus				Persistence of Tinnitus				Severity of Tinnitus			
			Univariable ANALYSIS		Multivariable Analysis		Univariable Analysis		Multivariable Analysis		Univariable Analysis		Multivariable Analysis	
			*p*-Value	OR (95% CI)	*p*-Value	OR (95% CI)	*p*-Value	OR (95% CI)	*p*-Value	OR (95% CI)	*p*-Value	OR (95% CI)	*p*-Value	OR (95% CI)
Age		1170	<0.001	1.1 (1,1.1)	0.12	(1,1.1)	<0.001	1.1 (1,1.1)	0.08	1 (1,1.1)	0.02	1.1 (1,1.2)	0.07	1.1 (1,1.3)
Sex	Male	916 (78.3)												
	Female	254 (21.7)	0.04	0.5 (0.3,1)	0.09	0.5 (0.3,1.1)	0.04	0.5 (0.3,1)	0.24	0.6 (0.3,1.4)	0.33	0.5 (0.1,2.1)		
Perceived stress	No	832 (71.1)												
	Yes	338 (28.9)	0.04	1.5 (1,2.3)	0.02 *	1.8 (1.1,2.9)	0.29	1.3 (0.8,2)			0.1	2.2 (0.8,5.8)	0.21	2.7 (0.6,13.1)
Smoking	None	68 (5.8)												
	Ex-smoker	616 (52.6)	0.1	0.5 (0.3,1.1)	0.01 *	0.3 (0.1,0.7)	0.06	0.5 (0.2,1)	0 *	0.3 (0.1,0.6)	0.05	0.2 (0.1,1)	0.06	0.1 (0,1.1)
	Current smoker	486 (41.5)	0.1	0.5 (0.3,1.1)	0 *	0.2 (0.1,0.6)	0.03	0.4 (0.2,0.9)	<0.001 *	0.2 (0.1,0.4)	0.1	0.3 (0.1,1.3)	0.04 *	0.1 (0,0.9)
Depression	No	1116 (95.4)												
	Yes	54 (4.6)	0.12	1.8 (0.8,4)	0.04 *	2.6 (1,6.7)	0.37	1.5 (0.6,3.6)			0	6.8 (2.1,21.6)	<0.001 *	132.7 (12.5,1409.6)
Hearing level	Mean hearing level (better ear)	-	<0.001	1.1 (1,1.1)			<0.001	1.1 (1,1.1)	0.55	1 (0.9,1)	<0.001	1.1 (1,1.1)	0.54	1 (0.8,1.1)
	Mean hearing level (worse ear)	-	<0.001	1 (1,1.1)			<0.001	1 (1,1.1)	0.17	1 (0.9,1)	<0.001	1.1 (1,1.1)	0.99	1 (0.9,1.1)
	Mean hearing level (better ear, high frequency)	-	<0.001	1 (1,1.1)			<0.001	1 (1,1.1)	0.8	1 (1,1.1)	<0.001	1.1 (1,1.1)	0.34	1.1 (0.9,1.2)
	Mean hearing level (worse ear, high frequency)	-	<0.001	1 (1,1.1)	<0.001 *	1.1 (1,1.1)	<0.001	1 (1,1.1)	<0.001 *	1.1 (1,1.1)	<0.001	1.1 (1,1.1)	0.18	1.1 (1,1.2)

* Statistically significant at *p* < 0.05.

**Table 4:** A 1:1 propensity score matching conducted to evaluate the effect of OSA risk on tinnitus by controlling the factors affecting tinnitus per the STOP-BANG questionnaire (experience, persistence, and severity of tinnitus).

Propensity Score Matching	Low-Risk Group (N = 861)	High-Risk Group (N = 309)	*p*-Value
**1. Experience of tinnitus**			
Model 1 (all combined)	33 (10.7%)	36 (11.7%)	0.798
Model 2 (perceived stress)	24 (7.8%)	36 (11.7%)	0.135
Model 3 (smoking)	21 (6.8%)	36 (11.7%)	0.052
Model 4 (depression)	20 (6.5%)	36 (11.7%)	0.036 *
Model 5 (high-frequency HL on better side)	36 (11.7%)	36 (11.7%)	1.000
**2. Persistence of tinnitus**			
Model 1 (all combined)	30 (9.7%)	33 (10.7%)	0.790
Model 2 (smoking)	19 (6.1%)	33 (10.7%)	0.060
Model 3 (high-frequency HL on better side)	34 (11.0%)	33 (10.7%)	1.000
**3. Severity of tinnitus**			
Model 1 (all combined)	0.29 ± 1.24	0.50 ± 1.58	0.071
Model 2 (smoking)	0.28 ± 1.23	0.50 ± 1.58	0.058
Model 3 (depression)	0.23 ± 1.00	0.50 ± 1.58	0.010 *

Abbreviations: HL, hearing loss. * Statistically significant at *p* < 0.05.

## Data Availability

The authors confirm that the data supporting the findings of this study are available within the article and its Supplementary Materials.

## Supplementary Materials

The following supporting information can be downloaded from https://www.mdpi.com/article/10.3390/jcm14217492/s1, Table S1. Tinnitus-associated covariates following univariate and multivariate logistic regression analyses.
