# Supplementary material for: Exploring a Possible Link Between Tinnitus and the Risk of Obstructive Sleep Apnea—A National Population-Based Cohort Study Using Propensity Score Matching Analysis
Source: J Clin Med. 2025 Oct 23;14(21):7492. doi: 10.3390/jcm14217492 (PMC12608285; doi:10.3390/jcm14217492)
Supplement: Supplementary file 1 [file jcm-14-07492-s001.zip › jcm-3897321-supplementary.docx]

| Variables | | Number  (%) | **Experience of tinnitus** | | | | **Prevalence of chronic tinnitus** | | | | **Severity of tinnitus** | | | |
| --- | --- | --- | --- | --- | --- | --- | --- | --- | --- | --- | --- | --- | --- | --- |
|  |  |  | Univariable Analysis | | Multivariable analysis | | Univariable Analysis | | Multivariable analysis | | Univariable Analysis | | Multivariable analysis | |
|  |  |  | *P*-value | OR  (95% CI) | *P*-value | OR  (95% CI) | *P*-value | OR  (95% CI) | *P*-value | OR  (95% CI) | *P*-value | OR  (95% CI) | *P*-value | OR  (95% CI) |
| Age | | 1170 | **< 0.001** | 1.1 (1,1.1) | 0.12 | (1,1.1) | **< 0.001** | 1.1 (1,1.1) | 0.08 | 1 (1,1.1) | **0.02** | 1.1 (1,1.2) | 0.07 | 1.1 (1,1.3) |
| Sex | Male | 916 (78.3) |  |  |  |  |  |  |  |  |  |  |  |  |
|  | Female | 254 (21.7) | **0.04** | 0.5 (0.3,1) | 0.09 | 0.5 (0.3,1.1) | **0.04** | 0.5 (0.3,1) | 0.24 | 0.6 (0.3,1.4) | 0.33 | 0.5 (0.1,2.1) |  |  |
| Income level | Lower | 107 (9.1) |  |  |  |  |  |  |  |  |  |  |  |  |
|  | Lower middle | 268 (22.9) | 0.69 | 0.9 (0.4,1.8) |  |  | 0.59 | 1.3 (0.5,3.1) |  |  | 0.99 | 1177400.8 (0,Inf) |  |  |
|  | Upper middle | 377 (32.2) | 0.63 | 0.8 (0.4,1.7) |  |  | 0.57 | 1.3 (0.5,3) |  |  | 0.99 | 9448158.9 (0,Inf) |  |  |
|  | Upper | 418 (35.7) | 0.59 | 0.8 (0.4,1.7) |  |  | 0.7 | 1.2 (0.5,2.8) |  |  | 0.99 | 3805884 (0,Inf) |  |  |
| Education level | Elementary school | 71 (6.1) |  |  |  |  |  |  |  |  |  |  |  |  |
|  | Middle school | 90 (7.7) | 0.85 | 1.1 (0.4,2.9) |  |  | 0.98 | 1 (0.4,2.6) |  |  | 0.87 | 0.8 (0,12.8) |  |  |
|  | High school | 464 (39.7) | 0.86 | 0.9 (0.4,2.1) |  |  | 0.59 | 0.8 (0.4,1.8) |  |  | 0.76 | 1.4 (0.2,11.1) |  |  |
|  | College | 545 (46.6) | **0.16** | 0.6 (0.2,1.3) | 0.96 | 1 (0.4,2.6) | **0.08** | 0.5 (0.2,1.1) | 0.55 | 0.7 (0.3,2) | 0.82 | 0.8 (0.1,6.6) |  |  |
| Marriage status | Yes | 1053 (90.0) |  |  |  |  |  |  |  |  |  |  |  |  |
|  | No | 117 (10.0) | 0.89 | 1 (0.5,1.9) |  |  | 0.66 | 0.8 (0.4,1.8) |  |  | 0.3 | 2 (0.6,6.9) |  |  |
| Subjective health perception | Good | 347 (29.7) |  |  |  |  |  |  |  |  |  |  |  |  |
|  | Normal | 650 (55.6) | 0.98 | 1 (0.6,1.6) |  |  | 0.92 | 1 (0.6,1.7) |  |  | 0.6 | 1.4 (0.4,5.4) |  |  |
|  | Poor | 173 (14.8) | **0.17** | 1.5 (0.8,2.7) | 0.43 | 1.3 (0.6,2.7) | **0.15** | 1.6 (0.8,3) | 0.22 | 1.6 (0.7,3.5) | **0.05** | 4.1 (1,16.7) | 0.07 | 9.7 (0.9,109.6) |
| BMI | | 24.8 ± 3.6 | 0.47 | 1 (1,1.1) |  |  | 0.29 | 1 (1,1.1) |  |  | 0.92 | 1 (0.9,1.1) |  |  |
| Hypertension | Normal | 510 (43.6) |  |  |  |  |  |  |  |  |  |  |  |  |
|  | Prehypertension | 365 (31.2) | 0.91 | 1 (0.6,1.6) |  |  | 0.57 | 1.2 (0.7,2) |  |  | 0.39 | 1.7 (0.5,5.6) |  |  |
|  | Hypertension | 295 (25.2) | 0.33 | 1.3 (0.8,2.1) |  |  | **0.08** | 1.6 (0.9,2.6) | 0.79 | 1.1 (0.6,2.2) | 0.22 | 2.1 (0.6,6.9) |  |  |
| Hypercholesterolemia | No | 825 (70.5) |  |  |  |  |  |  |  |  |  |  |  |  |
|  | Yes | 345 (29.5) | 0.76 | 1.1 (0.7,1.7) |  |  | 0.84 | 1.1 (0.7,1.7) |  |  | 0.29 | 1.7 (0.6,4.5) |  |  |
| Hypertriglyceridemia | No | 913 (78.0) |  |  |  |  |  |  |  |  |  |  |  |  |
|  | Yes | 257 (22.0) | 0.97 | 1 (0.6,1.6) |  |  | 0.96 | 1 (0.6,1.7) |  |  | **0.19** | 2 (0.7,5.4) | 0.51 | 1.7 (0.4,7.8) |
| Anemia | No | 1118 (95.6) |  |  |  |  |  |  |  |  |  |  |  |  |
|  | Yes | 52 (4.4) | 0.85 | 1.1 (0.4,2.8) |  |  | 0.96 | 1 (0.3,2.8) |  |  | 0.99 | 0 (0,Inf) |  |  |
| Perceived stress | No | 832 (71.1) |  |  |  |  |  |  |  |  |  |  |  |  |
|  | Yes | 338 (28.9) | **0.04** | 1.5 (1,2.3) | **0.02** | 1.8 (1.1,2.9) | 0.29 | 1.3 (0.8,2) |  |  | **0.1** | 2.2 (0.8,5.8) | 0.21 | 2.7 (0.6,13.1) |
| Alcohol consumption | none | 23 (2.0) |  |  |  |  |  |  |  |  |  |  |  |  |
|  | > 1/month | 715 (61.1) | **0.05** | 0.4 (0.1,1) | 0.21 | 0.5 (0.1,1.5) | **0.13** | 0.4 (0.1,1.3) | 0.21 | 0.4 (0.1,1.6) | 0.24 | 0.3 (0,2.3) |  |  |
|  | < 1/month | 432 (36.9) | **0.02** | 0.3 (0.1,0.9) | 0.08 | 0.3 (0.1,1.2) | **0.07** | 0.4 (0.1,1.1) | 0.12 | 0.4 (0.1,1.3) | 0.35 | 0.4 (0,3.1) |  |  |
| Average sleep time | | 7.1 ± 6.0 | 0.62 | 1 (1,1) |  |  | 0.82 | 1 (1,1) |  |  | 0.97 | 1 (0.9,1.1) |  |  |
| Smoking | None | 68 (5.8) |  |  |  |  |  |  |  |  |  |  |  |  |
|  | Ex-smoker | 616 (52.6) | **0.1** | 0.5 (0.3,1.1) | **0.01** | 0.3 (0.1,0.7) | **0.06** | 0.5 (0.2,1) | **0** | 0.3 (0.1,0.6) | **0.05** | 0.2 (0.1,1) | **0.06** | 0.1 (0,1.1) |
|  | Current smoker | 486 (41.5) | **0.1** | 0.5 (0.3,1.1) | **0** | 0.2 (0.1,0.6) | **0.03** | 0.4 (0.2,0.9) | **< 0.001** | 0.2 (0.1,0.4) | **0.1** | 0.3 (0.1,1.3) | **0.04** | 0.1 (0,0.9) |
| Masticatory difficulties | No | 913 (78.0) |  |  |  |  |  |  |  |  |  |  |  |  |
|  | Yes | 257 (22.0) | 0.2 | 1.3 (0.9,2.1) |  |  | 0.21 | 1.4 (0.8,2.2) |  |  | **0.19** | 2 (0.7,5.4) | 0.39 | 1.9 (0.4,8.5) |
| Asthma | No | 1139 (97.4) |  |  |  |  |  |  |  |  |  |  |  |  |
|  | Yes | 31 (2.6) | 0.63 | 0.7 (0.2,3) |  |  | 0.98 | 0 (0,Inf) |  |  | 0.99 | 0 (0,Inf) |  |  |
| Thyroid disease | No | 1146 (97.9) |  |  |  |  |  |  |  |  |  |  |  |  |
|  | Yes | 24 (2.1) | 0.53 | 1.5 (0.4,5) |  |  | 0.4 | 1.7 (0.5,5.8) |  |  | 0.29 | 3.1 (0.4,24.1) |  |  |
| Depression | No | 1116 (95.4) |  |  |  |  |  |  |  |  |  |  |  |  |
|  | Yes | 54 (4.6) | **0.12** | 1.8 (0.8,4) | **0.04** | 2.6 (1,6.7) | 0.37 | 1.5 (0.6,3.6) |  |  | **0** | 6.8 (2.1,21.6) | **< 0.001** | 132.7 (12.5,1409.6) |
| Otitis media | No | 1107 (94.6) |  |  |  |  |  |  |  |  |  |  |  |  |
|  | Yes | 63 (5.4) | **0.13** | 1.8 (0.9,3.7) | 0.18 | 1.7 (0.8,3.9) | **0.15** | 1.8 (0.8,3.8) | 0.31 | 1.6 (0.7,3.7) | 0.25 | 2.4 (0.5,10.7) |  |  |
| Renal disease | No | 1159 (99.1) |  |  |  |  |  |  |  |  |  |  |  |  |
|  | Yes | 11 (0.9) | 0.98 | 0 (0,Inf) |  |  | 0.98 | 0 (0,Inf) |  |  | 0.99 | 0 (0,Inf) |  |  |
| Hearing level | Mean hearing level (better ear) | - | **< 0.001** | 1.1 (1,1.1) |  |  | **< 0.001** | 1.1 (1,1.1) | 0.55 | 1 (0.9,1) | **< 0.001** | 1.1 (1,1.1) | 0.54 | 1 (0.8,1.1) |
|  | Mean hearing level (worse ear) | - | **< 0.001** | 1 (1,1.1) |  |  | **< 0.001** | 1 (1,1.1) | 0.17 | 1 (0.9,1) | **< 0.001** | 1.1 (1,1.1) | 0.99 | 1 (0.9,1.1) |
|  | Mean hearing level (better ear, high frequency) | - | **< 0.001** | 1 (1,1.1) |  |  | **< 0.001** | 1 (1,1.1) | 0.8 | 1 (1,1.1) | **< 0.001** | 1.1 (1,1.1) | 0.34 | 1.1 (0.9,1.2) |
|  | Mean hearing level (worse ear, high frequency) | - | **< 0.001** | 1 (1,1.1) | **< 0.001** | 1.1 (1,1.1) | **< 0.001** | 1 (1,1.1) | **< 0.001** | 1.1 (1,1.1) | **< 0.001** | 1.1 (1,1.1) | 0.18 | 1.1 (1,1.2) |
